# Chromosomal-level assembly of *Tokudaia osimensis, Tokudaia tokunoshimensis*, and *Tokudaia muenninki* genomes

**DOI:** 10.1038/s41597-023-02845-1

**Published:** 2023-12-21

**Authors:** Miki Okuno, Yuta Mochimaru, Kentaro Matsuoka, Takahiro Yamabe, Luisa Matiz-Ceron, Takamichi Jogahara, Atsushi Toyoda, Asato Kuroiwa, Takehiko Itoh

**Affiliations:** 1 Division of Microbiology, Department of Infectious Medicine, Kurume University School of Medicine, Kurume, Fukuoka, 830-0011 Japan; 2 School of Life Science and Technology, Tokyo Institute of Technology, Tokyo, 152-8550 Japan; 3 Reproductive and Developmental Science, Biosystems Science Course, Graduate School of Life Science, Hokkaido University, Sapporo, Hokkaido 060-0810 Japan; 4 Faculty of Law, Economics and Management, Okinawa University, Naha, Okinawa 902-0075 Japan; 5 Comparative Genomics Laboratory, National Institute of Genetics, Mishima, Shizuoka, 411-8540 Japan; 6 Advanced Genomics Center, National Institute of Genetics, Mishima, Shizuoka, 411-8540 Japan; 7 Division of Reproductive and Developmental Biology, Department of Biological Sciences, Faculty of Science, Hokkaido University, Sapporo, Hokkaido 060-0810 Japan

**Keywords:** Genome, Comparative genomics

## Abstract

Herein, we present the first high-quality long-read-based chromosome-level genome assemblies and gene annotations of the genomes of three endangered *Tokudaia* species: *Tokudaia osimensis*, *Tokudaia tokunoshimensis*, and *Tokudaia muenninki*. These species, which are endemic to different islands of the Ryukyu Islands, Japan, exhibited unique karyotypes and sex chromosomal characteristics. The genome assemblies generated using PacBio, Illumina, and Hi-C sequence data consisted of 13 (corresponded to 12 autosomes and one X chromosome), 23 (corresponded to 22 autosomes and one X chromosome), and 23 (corresponded to 21 autosomes and the neo- and ancestral X regions) chromosome-level scaffolds that contained 2,445, 2,477, and 2,661 Mbp of sequence data, respectively. Annotations of protein-coding genes were performed using RNA-Seq-based, homology-based, and Ab initio methods. BUSCO completeness values for every species exceeded 96% for genomes and 98% for genes. These data can be an important resource for contributing to our understanding of species genomes resulting from allopatric speciation and provide insights into mammalian sex-determination mechanisms and sex chromosome evolution.

## Background & Summary

**Fig. 1:**
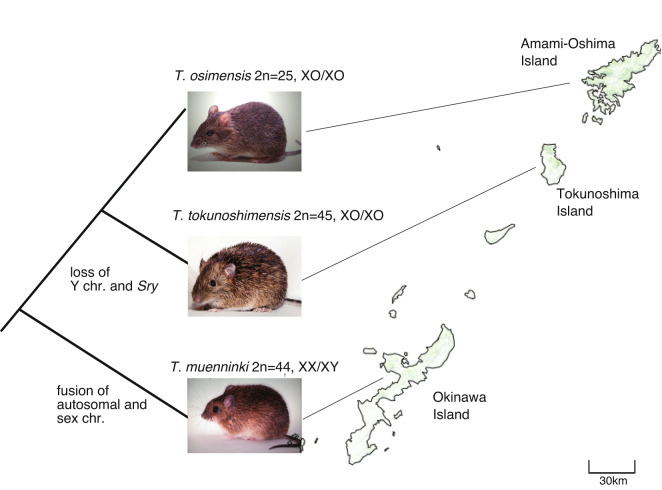
Habitat and phylogenetic relationships of three spiny rats. Karyotype information and evolutionary events assumed to have occurred in the ancestral species are shown. This map is based on a GSImap^74^ published by the Geospatial Information Authority of Japan. Photo courtesy: Kimiyuki Tsuchiya.

*Tokudaia* is a genus of murine rodents consisting of only three species of spiny rat, *Tokudaia osimensis*, *Tokudaia tokunoshimensis*, and *Tokudaia muenninki*, all of which have been listed as endangered because of their declining populations in recent years^1^. The three *Tokudaia* species are endemic to Amami-Oshima, Tokunoshima, and Okinawa Islands of the Ryukyu Islands, Japan. These islands were probably formed by repetitive sea regressions and transgressions among the Central Ryukyu Islands because of sea-level changes caused by glacial–interglacial cycles since the early Pleistocene^2^. Phylogenetic relationships between the three *Tokudaia* species correspond to the geographical distances between the three islands^3^. The three spiny rat species exhibit different karyotypes and unique sex chromosome characteristics. For example, the diploid number of chromosomes (2n) for both sexes in *T. osimensis* is 25, in *T. tokunoshimensis* is 45, and in *T. muenninki* is 44^4–6^. Moreover, both *T. osimensis* and *T. tokunoshimensis* have lost their Y chromosomes and *Sry*, a sex-determining gene in mammals^7^. Therefore, both males and females of these two species exhibit the XO/XO karyotype, with only one X chromosome;^4,5^ these species probably exhibit an *Sry*-independent sex-determination mechanism, different from that of common XX/XY-type mammals (Fig. 1). Various studies have been conducted to understand the sex-determination mechanism of these spiny rats, including experimental^8–10^ and genome sequence-based approaches. The results revealed that genes on the Y chromosome, other than *Sry*, have been conserved in the genome by translocation to the X chromosome^11,12^. Additionally, results showed a male-specific copy number variation (CNV) region upstream of *Sox9* on autosomal chromosomes, including the *Sox9* enhancer 14, which is involved in regulating *Sox9* and *Foxl2* expression, which are related to testicular differentiation^13^.

In contrast, *T. muenninki* exhibits an XX/XY sex chromosome configuration, similar to that of typical mammals; however, it contains a large neo-sex chromosome with autosomes, fused to the X and Y chromosomes^14,15^. A study showed that genes near the ancestral X and Y regions of the neo-X and neo-Y chromosomes accumulated male-specific mutations^15^. Unlike *T. osimensis* and *T. tokunoshimensis*, *Sry* has not been lost in *T. muenninki* and has multiple copies on the Y chromosome^3,16^. However, reportedly, *Sry* in *T. muenninki* is suspected to have lost its function as a sex-determining gene^9,17^. These three spiny rat species are suitable research subjects; however, their genome-based research has been limited to some RNA sequencing (RNA-Seq)-based^15,18^, fluorescence *in situ* hybridization-based^14,19^, and limited numbers of bacterial artificial chromosomes (BACs)-based^16^ studies, except for the *T. osimensis* genome, which has been studied by short-read sequencing^13^.

Herein, we provide the first report of high-quality, long-read-based chromosome-level genome assemblies and gene annotations for the three *Tokudaia* species. Our results can provide a valuable foundation for future studies regarding mammalian sex-determination mechanisms and sex chromosome evolution. Moreover, the constructed dataset may provide a valuable resource for the direct comparison of the genomes of species formed because of allopatric speciation over the last several million years.

## Methods

### Sample collection and sequencing

Whole-genome shotgun sequencing was performed using the PacBio and Illumina sequencing platforms. The genomic DNAs of *T. osimensis*, *T. tokunoshimensis*, and *T. muenninki* were isolated from the muscle tissues of male specimens that had accidentally perished (recovered in December 2005, December 2011, and March 2013, respectively) and had been stored in a deep freezer using the smart DNA prep Kit (Anlytik Jena, Jena, Germany). Genomic DNA from *T. osimensis* and *T. muenninki* was sheared into 30–100 kb fragments using a g-tube device (Covaris Inc., MA, USA). Although the DNA of *T. tokunoshimensis* was slightly degraded, this procedure was omitted. Subsequently, a continuous long-read (CLR) single-molecule real-time (SMRT) bell library was prepared using the SMRTbell Express Template Prep Kit 2.0 (Pacific Bioscience, CA, USA) according to the manufacturer’s instructions. Three CLR libraries were size-selected using the BluePippin system (Saga Science, MA, USA) with a lower cutoff of 40 kb. For each species, the library was run on two SMRT Cell 8Ms with Binding Kit 2.0 and Sequencing Kit 2.0. Through PacBio sequencing, 254.2 Gb, 272.1 Gb, and 271.8 Gb of CLRs were obtained for *T. osimensis*, *T. tokunoshimensis*, and *T. muenninki*, respectively (Table 1). Additionally, genomic DNA was fragmented to an average size of 500–600 bp using Focused-ultrasonicator M220 (Covaris Inc., MA. USA). Paired-end libraries with 450–550 bp insert sizes were constructed using the TruSeq DNA PCR-Free Library Prep kit (Illumina, CA, USA) and size-selected on an agarose gel using the Zymoclean Large Fragment DNA Recovery Kit (Zymo Research, CA. USA). The final libraries were sequenced following the 2 × 150 bp paired-end protocol for HiSeq 2500 (*T. osimensis*) and NovaSeq 6000 (*T. tokunoshimensis* and *T. muenninki*) systems (Illumina, San Diego, CA, USA). After Illumina sequencing, we obtained 448.1 Gb, 267.2 Gb, and 334.3 Gb of paired-end reads for *T. osimensis*, *T. tokunoshimensis*, and *T. muenninki*, respectively (Table 1). The Omni-C library was prepared using the Dovetail Omni-C Kit (Dovetail Genomics, Scott Valley, CA, USA) according to the manufacturer’s protocol. 1 × 10^6^ cultured cells were collected from the same *T. osimensis* individual from which the genomic DNA was extracted. The pulverized sample was processed into a proximity ligation library using the Omni-C Proximity Ligation Assay Mammalian Samples Protocol of the Omni-C Kit. The final library was sequenced using NovaSeq 6000 (Illumina) with a 2 × 150 bp read length, and 145.3 Gb of Omni-C reads were generated for *T. osimensis* (Table 1). The Arima Hi-C libraries were constructed from the same tissue (98.1 mg) of *T. tokunoshimensis* and cerebrum (177.4 mg) of the same *T. muenninki* individual from which genomic DNAs were extracted, using the Arima-HiC + Kit (Arima Genomics, CA, USA) according to the manufacturer’s instructions regarding Animal Tissues (A160132 v01) and library preparation (A160137 v00) using the TruSeq DNA PCR-Free Library Prep kit (Illumina, CA, USA). The obtained Hi-C libraries were run on the Illumina NovaSeq 6000 system with a 2 × 150 bp read length, and 153.8 Gb and 164.0 Gb of Arima Hi-C reads were generated for *T. tokunoshimensis* and *T. muenninki*, respectively (Table 1).

**Table 1:** Sequencing data used for three *Tokudaia* species genome assemblies.

Species	Library types	Platform	Reads number	Raw data (bp)	Average length (bp)	N50 length (bp)
*T. osimensis*	Illumina paired-end	HiSeq 2500	2,986.9 M	448.1 G	150	—
	PacBio CLR	Sequel II (CLR)	11.9 M	254.2 G	21,255	36,589
	Omni-C	NovaSeq 6000	968.7 M	145.3 G	150	—
*T. tokunoshimensis*	Illumina paired-end	NovaSeq 6000	1,781.3 M	267.2 G	150	—
	PacBio CLR	Sequel II (CLR)	13.2 M	272.1 G	20,591	37,487
	Arima Hi-C	NovaSeq 6000	1,025.6 M	153.8 G	150	—
*T. muenninki*	Illumina paired-end	NovaSeq 6000	2,228.8 M	334.3 G	150	—
	PacBio CLR	Sequel II (CLR)	12.0 M	271.8 G	22,648	38,431
	Arima Hi-C	NovaSeq 6000	1,093.2 M	164.0 G	150	—

### Genome size and heterozygosity estimation

**Fig. 2:**
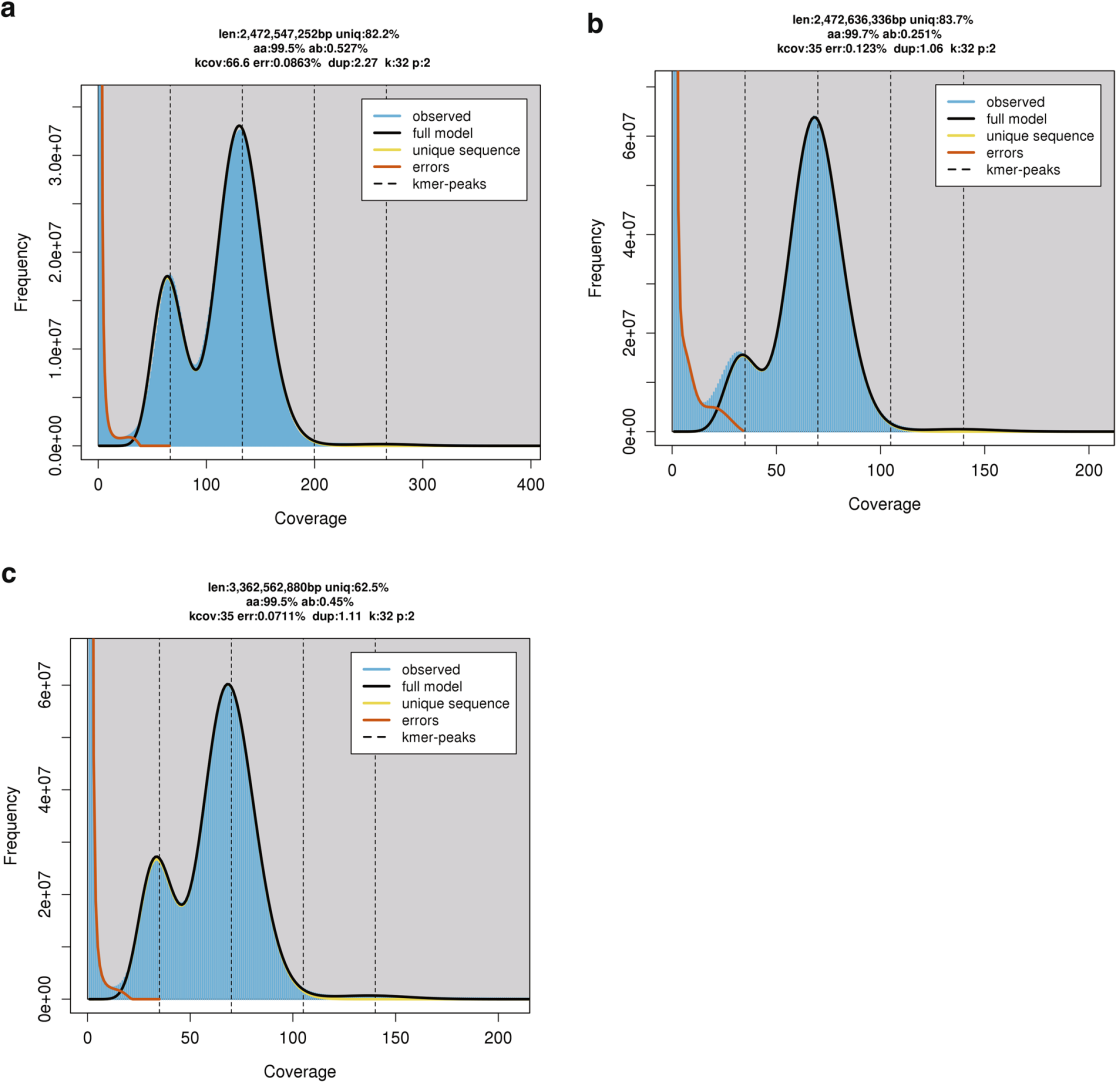
K-mer analysis of three *Tokudaia* species genomes. (**a**) *T. osimensis* (**b**) *T. tokunoshimensis* (**c**) *T. muenninki*. The estimated haploid genome sizes for *T. osimensis*, *T. tokunoshimensis*, and *T. muenninki* were 2,472.5 Mbp with 0.53% heterozygosity, 2,472.6 Mbp with 0.25% heterozygosity, and 3,362.6 Mbp with 0.45% heterozygosity, respectively. *T. muenninki*, with a genome size approximately 1 Gb larger than that of the other two species, shows consistency with a previous study demonstrating the presence of large heterochromatic blocks.

The genome size and heterozygosity of the three *Tokudaia* species were estimated using the Illumina sequencing data and a k-mer-based method. The sequenced reads from Illumina were filtered using Platanus_trim v1.0.7 (http://platanus.bio.titech.ac.jp/pltanus_trim) with default parameters. Using the trimmed reads, Jellyfish v2.3.0^20^ was applied to extract and count the canonical k-mers at k = 32. Following this, GenomeScope 2.0^21^ was used to estimate the haploid genome sizes and heterozygosities based on k-mer count data with parameters of “-k 32 -p 2” (Fig. 2). Thus, we estimated a haploid genome size of 2,472.5 Mbp with 0.53% heterozygosity for *T. osimensis*, 2,472.6 Mbp with 0.25% heterozygosity for *T. tokunoshimensis*, and 3,362.6 Mbp with 0.45% heterozygosity for *T. muenninki* (Fig. 2). The genome size estimation result that the *T. muenninki* has a genome size approximately 1 Gb larger than that of the other two species is consistent with a previous study showing that *T. muenninki* contains large heterochromatic blocks^14^.

### *De novo* genome assembly

The PacBio sequenced reads were used for genome assembly using Canu v2.1.1^22^, with the parameters of “corOutCoverage = 100 “batOptions = -dg 3 -db 3 -dr 1 -ca 500 -cp 50” -pacbio-raw”. The contigs were polished using two rounds of Arrow (Pacific Biosciences) and three rounds of NextPolish v1.4.0^23^. Haplotigs that were considered redundant because of the separate construction of both haplotypes were removed using Purge_Dups v1.2.5^24^. After removing redundant contigs, contigs with aberrant GC content (<2% or >98%), contaminant sequences derived from bacteria, parasitic organisms, such as *Toxoplasma*, and PacBio control sequences were removed based on the results of homology searches using BLASTN v2.11.0+^25^.

We performed Hi-C scaffolding using Hi-C datasets to obtain chromosome-level assemblies of the three *Tokudaia* species. The examination of input-purged assembly contigs before Hi-C scaffolding revealed the presence of sex chromosome-derived contigs that appeared to have been incorrectly removed by Purge_Dups. Therefore, Hi-C scaffolding was performed after reverting the sex chromosome-derived contigs, which were determined to have been erroneously deleted using the Hi-C read contact information. The Omni-C/Arima Hi-C reads were then mapped to the input contigs and processed to generate Hi-C contacts using Juicer v1.6^26^. Thereafter, the Hi-C contacts files were used for scaffolding by 3D-DNA v180922^27^ with the following parameter settings: “–editor-coarse-resolution 50000–editor-coarse-region 250000–input 1000”. We visualized the Hi-C contact map using Juicebox v1.11.08^28^, checked the mapping information of sequence reads on Integrated Genomics Viewer (IGV)^29^, and performed extensive manual curation to fix mis-assemblies, mis-scaffoldings, and mis-foldings caused by duplicated regions. Additionally, the mitochondrial genome sequences were constructed from the Illumina short reads of each individual using GetOrganelle v1.7.5.0^30^. All constructed mitochondrial sequences were circularized, and the obtained sequence lengths were 16,266, 16,263, and 16,250 bp for *T. osimensis*, *T. tokunoshimensis*, and *T. muenninki*, respectively.

We successfully constructed genomes of N50 = 234.0 Mbp, totaling 2,445.3 Mbp in *T. osimensis*; N50 = 125.1 Mbp, totaling 2,477.3 Mbp in *T. tokunoshimensis*; and N50 = 121.8 Mbp, totaling 2,660.8 Mbp in *T. muenninki* (Table 2). The length of constructed genomes of *T. osimensis* and *T. tokunoshimensis* were near their estimated genome sizes, 2,472.5 Mbp and 2,472.6 Mbp, respectively, whereas that of *T. muenninki* was only approximately 702 Mbp less than its estimated genome size of 3,362.6 Mbp. It was assumed to be because of the inability to construct the sequences of the heterochromatin region, which are highly repetitive. In *T. osimensis*, 99.79% of the assembled contigs were incorporated into 13 scaffolds, which corresponded to 12 autosomes and one X chromosome, suggesting that all 2n = 25 chromosomal karyotypes were constructed. Similarly, in *T. tokunoshimensis*, 99.87% of the assembled contigs were incorporated into 23 scaffolds, which corresponded to 22 autosomes and one X chromosome, suggesting that all 2n = 45 chromosomal karyotypes were constructed. Conversely, in *T. muenninki*, 91.31% of the assembled contigs were incorporated into 23 scaffolds, which were considered to correspond to 21 autosomes and the neo- and ancestral X regions in *T. muenninki*, with 2n = 44. For the ancestral Y region, the orientation and order of the candidate contigs could not be determined through Hi-C scaffolding, and chromosome-level scaffolds were not constructed. Therefore, sequences derived from the ancestral Y region were constructed as unplaced scaffolds. The unplaced scaffold accounted for 8.69% (231.3 Mbp) of the entire genome sequence, suggesting that it contained sequences of the heterochromatin region in addition to those of the ancestral Y region.

**Table 2:** Assembly statistics of three *Tokudaia* species genomes.

	*T. osimensis*	*T. tokunoshimensis*	*T. muenninki*
	2n = 25, XO/XO	2n = 25, XO/XO	2n = 44, XX/XY
Genome assembly statistics			
#Scaffolds	124	160	1,528
#Chromosome-level scaffolds	13	23	23
#unplaced scaffolds	110	136	1,504
#mitochondrial DNA contig	1	1	1
Total scaffold length (bp)	2,445,315,397	2,477,310,555	2,660,819,200
Total Chr.-level scaffold length (bp)	2,440,098,537	2,473,996,358	2,429,465,518
Anchored to chromosome (%)	99.79	99.87	91.31
Longest scaffold (bp)	269,377,760	179,766,896	175,831,743
Contig N50 (bp)	8,462,634	13,597,559	19,740,843
Contig L50	88	57	34
Scaffold N50 (bp)	234,036,378	125,076,325	121,843,864
Scaffold L50	5	9	10
Gaps (bp)	270,541	205,000	125,000
BUSCO evaluation (v5.4.7, genome mode, glires_odb10)			
Complete BUSCOs (%)	96.15	96.14	96.30
single-copy BUSCOs (%)	94.97	94.81	95.20
duplicated BUSCOs (%)	1.18	1.33	1.10
Fragmented BUSCOs (%)	0.74	0.82	0.64
Missing BUSCOs (%)	3.11	3.04	3.06
Merqury evaluation			
Completeness	93.71	93.72	93.14
QV	45.21	47.85	45.32

**Fig. 3:**
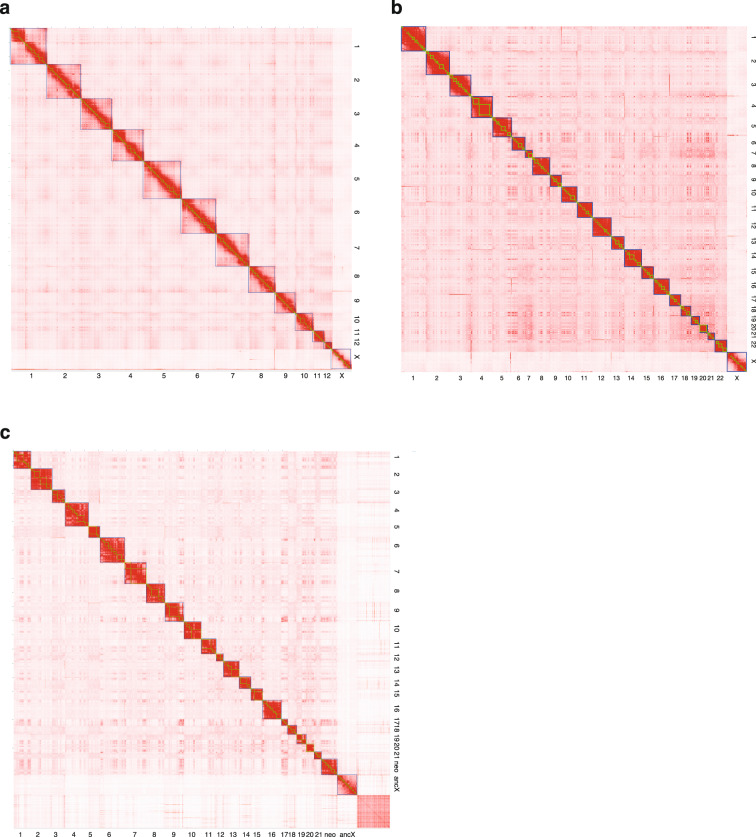
Genome-wide Hi-C contact map of three *Tokudaia* species genomes. The blue squares represent chromosomes. (**a**) Hi-C contact map of *T. osimensis* comprising 13 chromosome-level scaffolds (representing 12 autosomes and the X chromosome) (**b**) Hi-C contact map of *T. tokunoshimensis* comprising 23 chromosome-level scaffolds (representing 22 autosomes and the X chromosome) (**c**) Hi-C contact map of *T. muenninki* comprising 23 chromosome-level scaffolds (representing 21 autosomes and the neo- and ancestral X regions).

The chromosome numbers and orientations of the scaffolds were determined based on the synteny relationship examined among the three constructed chromosome-scale scaffolds and mouse chromosomes by extracting the best bidirectional alignments using minimap2^31^ and corresponding them to the results of previous chromosome painting studies^14,19^. Figure 3 illustrates the Hi-C contact map constructed using the rearranged sequences. In all three species, the X chromosome had half of the coverage; therefore, the contacts were weak, whereas in *T. muenninki*, the fragmented Y chromosome-derived sequences were clustered in the lower-right corner of the contact map as unplaced scaffolds.

### Gene structure prediction and functional annotation

Gene structures were predicted using the following methods: (1) an RNA-Seq-based method that predicts gene structures on the basis of transcriptome sequencing results, (2) a homology-based method that predicts gene structures on the basis of protein sequences of related species, and (3) an Ab initio method that learns the features of gene regions and predicts them directly from the genome sequence. The prediction results of the three methods were integrated using an integration tool. The three prediction methods are described below.

1. RNA-Seq-based method
  For the RNA-Seq-based method, gene prediction was performed using both mapping and *de novo* methods using RNA-Seq data (*T. osimensis*: DRP009149^32^, *T. tokunoshimensis*: DRP010494^33^, and *T. muenninki*: DRP003435^34^ and DRP004135^35^). In the mapping-based method, HISAT2 v. 2.2.1^36^ was used to map RNA-Seq reads to genomic sequences, and the mapped reads were assembled using StringTie v2.2.0^37^. Next, *de novo* methods were used to assemble the RNA-Seq reads using the transcript assemblers Trinity v2.12.0^38^ and Oases v2.09^39^. Redundant sequences were removed from the assembled contigs using CD-HIT v4.8.1^40^ and the contigs were aligned to the genomic sequence using GMAP v2015–09–29^41^. Finally, open reading frames were determined using both mapping and *de novo* methods using TransDecoder (https://github.com/TransDecoder/TransDecoder) to predict gene structures.
2. Homology-based method
  The protein sequences of eight mouse subfamily (Murinae) species annotated in NCBI RefSeq, *Mus musculus*^42^, *Mus caroli*^43^, *Mus pahari*^44^, *Rattus norvegicus*^45^, *Rattus rattus*^46^, *Arvicanthis niloticus*^47^, *Grammomys surdaster*^48^, *Mastomys coucha*^49^ were downloaded. They were splice-aligned to the genome sequence using Spaln v2.3.3^50^ to predict gene structures.
3. Ab initio-based method

Augustus v3.3^51^ was used to predict the gene structure. First, 1,000 randomly selected genes from the RNA-Seq-based predicted genes were selected to train the gene model, which was further used to predict the gene structure.

The results predicted by the three methods were integrated into the GINGER pipeline^52^. Additionally, predicted genes that satisfied all the requirements of the confidential *M. musculus*^42^ or *R. rattus*^46^ full-length homology-based results were extracted and integrated into the GINGER results. The requirements were as follows: Compared with mouse or rat orthologous genes, predicted genes should have (a) 80% or more identity and sequence coverage, (b) less than 3-amino acid length differences with all exons, and (c) all introns should be 0.5 to 2.0 times the length of the corresponding introns of the mouse or rat. If an exon overlapped between the newly predicted exons and the GINGER exon after integration, the exon with a longer CDS was used for the final result.

The statistics for the predicted gene sets are listed in Table 3. A total of 22,233, 22,106, and 22,240 protein-coding genes were predicted in *T. osimensis*, *T. tokunoshimensis*, and *T. muenninki*, respectively. These numbers were similar to those annotated in the closely related species mouse (22,173) and rat (22,215). Other statistics were similar to those for the mice and rats. Subsequently, the homology information of the mouse gene was assigned to the predicted genes as functional annotation. A homology search was performed using BLASTP v2.11.0+^25^ against the mouse RefSeq sequence^42^, and if a hit with more than 80% identity and coverage was found, the mouse annotation information was assigned to the corresponding predicted gene. Consequently, 18,469, 18,354, and 18,471 genes, which corresponded to approximately 83% of the total genes, were annotated for *T. osimensis*, *T. tokunoshimensis*, and *T. muenninki*, respectively.

**Table 3:** Gene annotation statistics of three *Tokudaia* species genomes.

	*T. osimensis*	*T. tokunoshimensis*	*T. muenninki*	*(M. musculus)*	*(R. norvegicus)*
Predicted genes statistcs					
#Gene	22,233	22,106	22,240	22,173	22,215
#Single exon genes	3,114	2,985	2,983	3,766	4,073
Total exon + intron length (bp)	825,581,176	832,857,796	823,145,008	848,421,758	843,812,967
Mean exon + intron length (bp)	37,133.1	37,675.6	37,011.9	38,263.7	37,983.9
#Exons	199,034	198,638	199,682	201,287	200,148
#Exons per gene	8.95	8.99	8.98	9.08	9.01
Total exon length (bp)	35,267,016	35,143,527	35,354,169	36,662,602	36,311,498
Mean exon length (bp)	177.2	176.9	177.1	182.1	181.4
#CDSs	22,233	22,106	22,240	22,173	22,215
Total CDS length (bp)	35,267,016	35,143,527	35,354,169	36,662,602	36,311,498
Mean CDS length (bp)	1,586.3	1,589.8	1,589.7	1,653.5	1,634.6
#Intron	176,801	176,532	177,442	179,114	177,933
Total intron length (bp)	790,314,160	797,714,269	787,790,839	811,759,156	807,501,469
Mean intron length (bp)	4,470.1	4,518.8	4,439.7	4,532.1	4,538.2
%GT–AG splice sites	99.04	99.04	99.02	98.95	98.09
BUSCO evaluation (v5.4.7, protein mode, glires_odb10)					
Complete BUSCOs (%)	98.70	98.80	99.01	99.47	98.76
single-copy BUSCOs (%)	98.08	98.01	98.36	98.63	97.23
duplicated BUSCOs (%)	0.62	0.80	0.65	0.84	1.53
Fragmented BUSCOs (%)	0.32	0.30	0.20	0.16	0.36
Missing BUSCOs (%)	0.98	0.90	0.79	0.37	0.88

## Data Records

Genomic sequencing data (Illumina, PacBio, Hi-C) are available in the NCBI SRA database under BioProject ID PRJDB16410. The accession numbers of the Illumina sequencing data are DRR066821 and DRR066822^53^ (*T. osimensis*), DRR495707^54^ (*T. tokunoshimensis*), and DRR495711^55^ (*T. muenninki*). The accession numbers of the PacBio sequencing data are DRR495851^56^ and DRR495852^57^ (*T. osimensis*), DRR495706^58^ and DRR495709^59^ (*T. tokunoshimensis*), and DRR495710^60^ and DRR495713^61^ (*T. muenninki*). The accession numbers of the Hi-C sequencing data are DRR378863^62^ (*T. osimensis*), DRR495708^63^ (*T. tokunoshimensis*), and DRR495712^64^ (*T. muenninki*). The accession numbers of the assemblies are BTPL01000001– BTPL01000123^65^ (*T. osimensis*), BTHU01000001–BTHU01000159^66^ (*T. tokunoshimensis*), and BTHS01000001–BTHS01001527^67^ (*T. muenninki*). The accession number of mitochondrial genomes are LC778283.1^68^ (*T. osimensis*), LC778284.1^69^ (*T. tokunoshimensis*), and LC778282.1^70^ (*T. muenninki*). The genome annotation files are available in the Figshare database^71^.

## Technical Validation

### DNA and RNA quality

Agarose gel electrophoresis was used to assess the quality of the extracted DNA. The main band was >20 kb, and the DNA spectrophotometer ratio (260 nm/280 nm) was >1.8. The quality of the purified RNA molecules was examined by 2100 Bioanalyzer (Agilent Technologies, Santa Clara, CA, USA), and RNA integrity (RIN) was >7.0.

### Assembly evaluation

The quality and completeness of the chromosome assembly were evaluated using two independent approaches. First, the QV value and completeness were estimated using Merqury v1.3^72^ by comparing k-mers in the assembly with those found in the Illumina sequence reads. The results revealed that QV values of the assembly were 45.21, 47.85, and 45.32 for *T. osimensis*, *T. tokunoshimensis*, and *T. muenninki*, whereas completeness values were 93.71%, 93.72%, and 93.14%, respectively. Second, the completeness of the assembly was assessed using BUSCO v5.4.7^73^ (genome mode) with 13,798 single-copy orthologs from the glires_odb10 database. The analysis revealed that 96.15%, 96.14%, and 96.30% of the complete BUSCOs were identified in the genomes of *T. osimensis*, *T. tokunoshimensis*, and *T. muenninki*, respectively.

### Gene annotation evaluation

The completeness of the annotated protein-coding genes was assessed using BUSCO v5.4.7^73^ (protein mode) with 13,798 single-copy orthologs from the glires_odb10 database. The analysis revealed that 98.70%, 98.80%, and 99.01% of the complete BUSCOs were identified in the annotated genes of *T. osimensis*, *T. tokunoshimensis*, and *T. muenninki*, respectively.

## Data Availability

No custom code was used in this paper, and all of the programs used were publicly available.
